# Cellular, Molecular, Pharmacological, and Nano-Formulation Aspects of Thymoquinone—A Potent Natural Antiviral Agent

**DOI:** 10.3390/molecules28145435

**Published:** 2023-07-16

**Authors:** Ambreen Shoaib, Shamama Javed, Shadma Wahab, Lubna Azmi, Mohammad Tabish, Muhammad H. Sultan, Karim Abdelsalam, Saad S. Alqahtani, Md Faruque Ahmad

**Affiliations:** 1 Department of Clinical Pharmacy, College of Pharmacy, Jazan University, Jazan 45142, Saudi Arabia; kaboelsoud@jazanu.edu.sa; 2 Pharmacy Practice Research Unit (PPRU), College of Pharmacy, Jazan University, Jazan 45142, Saudi Arabia; 3 Department of Pharmaceutics, College of Pharmacy, Jazan University, Jazan 45142, Saudi Arabia; sjahmad@jazanu.edu.sa (S.J.); mhsultan@jazanu.edu.sa (M.H.S.); 4 Department of Pharmacognosy, College of Pharmacy, King Khalid University, Abha 62529, Saudi Arabia; sabdulwahab@kku.edu.sa; 5 Department of Pharmaceutical Chemistry, Institute of Pharmaceutical Sciences, University of Lucknow, Lucknow 226007, India; azmilubna@gmail.com; 6 Department of Pharmacology, College of Medicine, Shaqra University, Shaqra 11961, Saudi Arabia; tabish@su.edu.sa; 7 Department of Clinical Pharmacy, College of Pharmacy, King Khalid University, Abha 62529, Saudi Arabia; ssalqahtani@jazanu.edu.sa; 8 Department of Clinical Nutrition, College of Applied Medical Sciences, Jazan University, Jazan 45142, Saudi Arabia

**Keywords:** clinical trial, immunity, mechanism, nanotechnology, thymoquinone, virus

## Abstract

The goal of an antiviral agent research is to find an antiviral drug that reduces viral growth without harming healthy cells. Transformations of the virus, new viral strain developments, the resistance of viral pathogens, and side effects are the current challenges in terms of discovering antiviral drugs. The time has come and it is now essential to discover a natural antiviral agent that has the potential to destroy viruses without causing resistance or other unintended side effects. The pharmacological potency of thymoquinone (TQ) against different communicable and non-communicable diseases has been proven by various studies, and TQ is considered to be a safe antiviral substitute. Adjunctive immunomodulatory effects in addition to the antiviral potency of TQ makes it a major compound against viral infection through modulating the production of nitric oxide and reactive oxygen species, decreasing the cytokine storm, and inhibiting endothelial dysfunction. Nevertheless, TQ’s low oral bioavailability, short half-life, poor water solubility, and conventional formulation are barriers to achieving its optimal pharmacologic benefits. Nano-formulation proposes numerous ways to overcome these obstacles through a small particle size, a big surface area, and a variety of surface modifications. Nano-based pharmaceutical innovations to combat viral infections using TQ are a promising approach to treating surmounting viral infections.

## 1. An Introduction to Thymoquinone and Viral Diseases

Viral diseases are a serious concern worldwide, because of the extreme danger and complexity they pose to human life, due to their ability to cause a rapid outbreak among developing nations [1]. Viruses are intracellular parasites that encompass one of the significant classes of pathogens [2]. Viruses can generate several diseases such as: acquired immunodeficiency syndrome, dengue, Middle East respiratory syndrome, polio, etc. [3,4]. Viruses infect healthy cells and cause apoptosis in infected cells. Among all of these viruses, a small proportion of them cause the depletion of immune cells and, consequently, deteriorate the host’s immune system [5,6].

Developing remedies for viral diseases presents a tremendous challenge due to the easy transformation of viruses, the resistance of viral pathogens, new viral strain developments, side effects, the high cost of medicines, and the development of resistance [7]. The standard therapy for viral infections includes destroying the target pathogen; instead, many therapies inhibit viral development and shorten the length of the disease [8]. In modern times, the emergence of resistance of microbial and viral pathogens against common antimicrobial and antiviral drugs, mostly for respiratory tract infections (RTI), has increased day by day, which is a significant health problem affecting the treatment of these conditions [8]. According to the WHO’s (World Health Organization)’s current list, viruses, especially RNA viruses, are among the top ten health threats globally. The list includes: the influenza pandemic, human immunodeficiency virus, dengue virus, Ebola virus (EBOV), and SARS-COV-2. Other pathogens include Zika virus, various hemorrhagic fevers, Nipah virus, Middle East respiratory syndrome, SARS-COV-2, SARS-CoV, and disease X [9,10,11,12].

Plants have long been used to prevent disease. Botanicals are used to treat around eighty percent of the world’s population. Alternative medicine shows the economic importance of plants [13]. Natural medicinal plants are an essential source of different bioactive and therapeutic compounds with antiviral activities; very few studies have been conducted on various medicinal plants’ antiviral potential [14,15]. Since ancient times, the use of various medicinal plants has been considered to treat human diseases. A medicinal herb can change the pathological and physiological processes used to prevent and treat disease. Recently, there has been a remarkable increase in the therapeutic use of medicinal plants for different diseases, compared with chemical and synthetic drugs, because of their ease of availability without requiring a prescription, less interference from healthcare professionals, their low cost, and the belief that they are associated with fewer adverse effects [16,17]. Natural spices and herbs have been used since time immemorial for their medicinal and therapeutic purposes. These natural therapies are used in the treatment of multiple diseases around the globe. Barrett et al. reported that fifty percent of Americans use herbal and natural remedies to treat ailments [18,19].

*N. sativa* (Ranunculaceae), also known as black seed, is an herb that is commonly used in the Middle East as a natural food and traditional medicine [20]. Some research and data suggest that black seed and its principal active constituent TQ has significant antioxidant and cytoprotective effects against organ damage. It has antifungal, antiviral, anti-allergic, analgesic, anti-tumor, and antipyretic activity [18,21]. *N. sativa* oil has been shown to lower viral load in mice infected with cytomegalovirus to an undetectable level in the spleen and liver within ten days after being administered via the intraperitoneal route. This may be due to increasing the function and number of CD T cells and interferon-gamma production [22,23]. In patients (*n* = 30) infected with HCV (hepatitis C virus), in whom IFN-*α*/ribavirin drug therapy was contraindicated, a significant improvement was observed in HCV viral load. Moreover, various laboratory parameters have been shown to improve with *N. sativa* seed oil, including red blood cells, platelet count, and total protein, along with a decrease in postprandial glucose and fasting blood glucose in both non-diabetic and diabetic HCV patients [22,24]. In a case reported by Onifade et al., an HIV-positive patient was treated with 10 mL of *N. sativa* seed oil twice a day for six months, and complete seroconversion and regaining of physical health were reported [25]. In another case, a 27-year-old female patient was diagnosed with HIV infection during her antenatal care. She was not able to take antiretroviral therapy, so herbal medicine therapy was started with honey and black cumin mixture at a dose of 10 mL TID for a year, at which time, serology testing revealed an undetectable viral load [22,26].

One study showed that *N. sativa* black seed oil prevented rodent cytomegalovirus infection in experimental models. [27]. This may show that *N. sativa* seed oil and its active constituent TQ have a potent antiviral effect against murine cytomegalovirus infection, which may be due to the upregulation of IFN-g and macrophage activation [28]. It could be used as a potential co-therapeutic agent against numerous conditions related to immunodeficiency [6]. Currently, COVID-19, HIV/AIDS, and other viral diseases are serious global threats. Therefore, TQ derived from *N. sativa* might be an excellent natural product to cure these infectious diseases. Thus, this review focused more on the new technologies needed to achieve the full pharmacological potential of TQ. To do this, its poor oral bioavailability, short half-life, poor water solubility, and traditional formulation restrictions must be overcome. Numerous strategies for overcoming these challenges have been proposed using nano-formulations, including the use of extremely small particles, a large surface area, and various surface changes. In order to overcome viral infections, nano-based pharmaceutical advancements using TQ show great promise.

## 2. The Immune Mechanism in Viral Disease

When a virus comes in contact with the human body, it invades cells to survive and replicate, causing infection. The cells utilize histocompatibility complex proteins that lead to protein fragmentation inside the cell as well as on the cell membrane [29]. Once the cells are infected, these kinase cluster forms encompass virus-made protein components that can affect different inflammatory biomarkers, especially cytokines, along with macrophages such as neutrophils. These then release diverse compounds that cause cell disruption, and the production of cationic proteins, cytotoxic cytokines, lipid mediators, metalloprotease, and oxygen-release sources [30]. An accumulation of free radicals in mitochondria can lead to cellular damage [31], which plays an essential role in the mechanism of viral diseases and their prevention.

The cytotoxic T cell is a particular type of immune cell found in the human body that destroys virus-loaded cells that produce toxic mediators [32]. Several specialized proteins (T cell receptors) are found on the surface/membrane of cytotoxic T lymphocytes, and their basic function is to recognize the infected cells. There are T cell receptors on each cytotoxic cell that recognize a specific antigenic peptide attached to an important histocompatibility complex protein as soon as the pathogenic peptide can detect the T cell receptor, which may alert the T cell to the infection [33]. Cytotoxic factors are released by T cells that are usually stored inside compartments termed granules. The factors kill the infected cell, trigger their release, and prevent the survival of the spreading virus [34]. A particular protein also acts as a mediator, i.e., perforin, and the initial function of this protein is that it can make cell membrane pores. The pores on the surface may facilitate the destruction of the cell, with the help of other factors, into a target cell [31]. Granzymes (a form of enzyme) are also released and stored in the granules. They enter into the target cells via the pores developed by perforin. The cytokines released during viral infection are type I interferons that mediate the stimulation of either the immune response or the subsequent formation of responsive tolerance towards viruses [33].

Accordingly, viruses are highly adaptable, and they have developed different ways to avoid recognition by T cells. However, other specialized cells have a smaller number of major histocompatibility complex proteins on the surface and they kill the virally infected cells by releasing a toxic substance. The death of contaminated cells through CD8+ T cells seems to be the leading cause of liver failure in certain non-cytopathic viral diseases, like hepatitis B and C viruses [35]. Antiviral defense machines are stimulated when the immune system recognizes a viral infection [36]. Few pathogens have innate characteristics that make immunity regulation impossible, and even efforts by the host defense response at regulation result in significant tissue injury [37].

The interaction of immunoglobulin G with the fragment crystallizable receptors onto proinflammatory cytokines leads to the activation of inflammatory mediators, and reactions to antibody-mediated chronic inflammation result in toxins [38]. Viruses like the respiratory syncytial virus have antigens that really can trigger the immunoglobulin E reaction including type I oversensitivity [39]. The T cell is unknowing that the contaminated cell accommodates a virus. A natural killer cell is a type of immune killer specializing in killing cells with fewer MHC proteins on their membrane [40]. Whenever a natural killer cell determines the existence of a cell that has less MHC than usual, it produces toxins, close to how cytotoxic T cells destroy virus-infected cells [41]. Site pattern-recognition receptors which are specifically regulated through the immune system, including macrophages, may be identified as DNA and RNA formed through viral content-contaminated cells [42].

The Toll-like receptors, retinoic acid persuade genetic material, and NOD-resembling receptors are one of the many forms of innate immune receptors that can engage throughout the immune reaction [31,42]. Endocytic Toll-like receptors like TLR_3_, TLR_7_, TLR_8_, and TLR_9_ respectively identify nucleic acids of the virus and its double-stranded shaped RNA intermediate products and are normally activated by virus infections [43]. The NLRs accept viral DNA sequences, while the intracellular retinoic acid-inducible gene-like responders identify gene structure RNA or RNA formed by genomic DNA [44]. The sequence with an innate immune response that takes place when a virus enters the body can influence the infection’s result. natural killer (NK) cells, dendrite cells (DCs), and macrophages are examples of innate cells that develop anti-inflammatory activity like interleukin-10 represents as transforming growth factor (TGF), and IL-10 [44].

## 3. Antiviral Properties and Mechanisms of Action of Thymoquinone in Cellular and Molecular Aspects

### 3.1. Antiviral Properties

TQ is a phytoconstituent having different pharmacological actions along with all emphasis is placed on its antiviral activity. This natural product shows its action as an antiviral agent by decreasing inhibition of viral replication of the virus [45]. It has a high therapeutic index for its target [46]. The other hybrid product of TQ shows synergistic effects among linked pharmacophores [47]. An in vitro model of a TQ–artesunic acid hybrid shows considerably higher antiviral activity from the standard drugs, artesunic acid and ganciclovir [46]. Correspondingly it also exhibits synergistic antiviral activity when co-administered with other phytoconstituents, such as curcumin. Curcumin and TQ show effective actions against a murine cytomegalovirus infection model and avian influenza virus (H9N2 AIV) [48]. A recent study recommended that it modulates IL-8 secretion, tryptophan repressor gene expression, and viral load in coronavirus infections [49]. Furthermore, examination of gene expression reveals a decrease in viral loads following treatments, which in turn affects viral survival within the cell [49]. TQ is undergoing clinical trials for the treatment of infection related to the hepatitis C virus [24].

**Figure 1:**
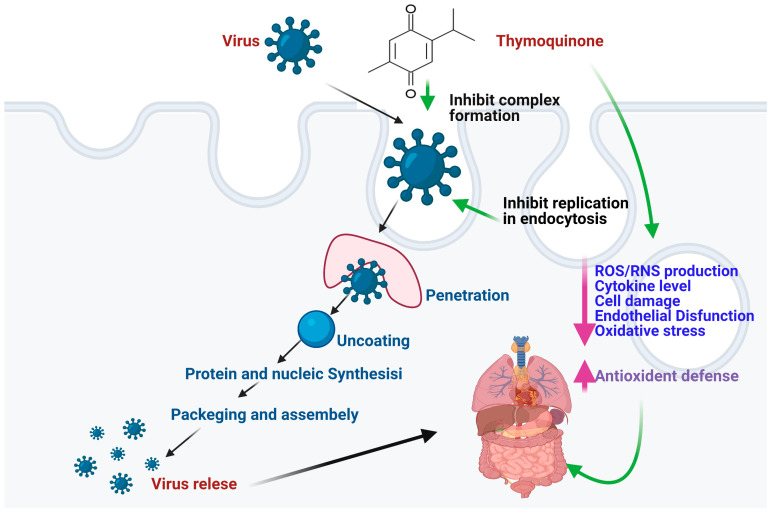
A schematic representation of the Antiviral mechanism of action for Thymoquinone (TQ).

The potential therapeutic value of TQ in the treatment of SARS-COV-2 has attracted more attention in recent years due to its medical significance. The compound TQ possesses antiviral activity against the coronavirus infection as it has the probable binding at SARS-CoV-2: angiotensin-converting enzyme-2 (ACE-2) interface, and consequently could be predicted as a persuasive inhibitor to interfere with viral-host interactions [50]. This compound can be utilized through the human cell-surface receptor HSPA5 and shows positive action on the virus and thus can be used in patients with high risk to reduce the hazard of SARS-COV-2 [51]. TQ interface binds with the key residues and may dislocate host recognition, which may cure the viral infection. It also inhibits the murine cytomegalovirus replication in infected mice which might be facilitated by an increase in the number and function of macrophages and the production of interferon-gamma [6]. Some studies show that the Black seeds (*N. sativa)* and their pharmacologically active constituent TQ show potent antiviral mechanisms by inhibiting the proliferation of viruses or by its immunomodulatory effect. Consequently, we have to go through the mechanism of action for a better understanding through which it shows its antiviral action (Figure 1).

### 3.2. Mechanisms of Action

TQ is a temperature-sensitive phytoconstituent and hydrophobic too; resulting in poor bioavailability due to its hydrophobicity and low solubility [52]. However, dosage form other than conventional (nano-formulations) increases the efficacy and bioavailability that unveiled remarkable immunomodulatory and antiviral activities at a specific dose [53]. Numerous pharmaceutical formulation research has focused on it because its hydrogel preparations are biologically friendly and provide the continuous release of medicines [54]. It shows its antiviral activity by regulating ROS and NO production [55]. It also reduces cytokine storm-mediated endothelial dysfunction. It also shows inhibitory effects on viral infection and improved multiple organ dysfunction syndrome complications by fixing the redox and immune balances [50]. This is perhaps via the redox mechanism, which may reduce the inflammatory response and systemic oxidative stress. Consequently, TQ reduces the early stage inflammatory biomarkers viz., endothelial cell-specific molecule 1, C-reactive protein, and vascular endothelial growth factor [53]. Remarkably, inflammatory cytokines like- tumor necrosis factor-α (TNF-α), Interleukin-1α, Interleukin 2, Interleukin-6, and Interleukin-10 also augment the inducible nitric oxide synthase-mediated NO production [50].

Besides ROS generation, inflammatory cytokine production by activated phagocytes as a result of the activation of nuclear factor kappa-light-chain-enhancer of activated B cells (NF-B) also contributes to the oxidative stress associated with virus-induced phagocyte activation. Inhibition of NF-κB can thus suppress inflammatory genes, halt the cytokine storm, and reduce immune cell invasion and activation, protecting tissue and organs from injury [56]. Docking studies suggest that TQ may inhibit SARS-CoV-2 replication by interfering with viral binding to ACE-2 receptors. As a result, it is able to block the virus from entering the host cell and from replicating within the host cell and act as a potent antiviral agent [57,58].

## 4. Pharmacological Applications of Thymoquinone as an Antiviral Agent

**Figure 2:**
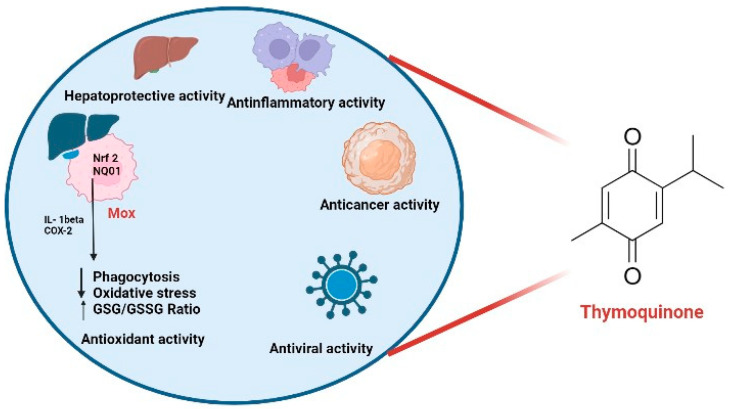
The use of thymoquinone in multiple applications for the treatment of a variety of disorders. The symbol ↑ shows an increase whereas the symbol ↓ denotes a decline in that particulars.

Viral infections can induce apoptotic cell death and reduce host lymphocyte counts. Antioxidants have the potential to inhibit the apoptosis induced by the virus along with they also prevent the replication of the virus in target cells [59]. Therefore, it can be concluded that the antiviral and antioxidant possessions can be linked with each other and that could be a potential source to surmount viral infection [60]. TQ is the active constituent of many plants but is widely found in *N. sativa* and has different valuable properties [21]. When we focus on the TQ exhibits a broad-spectrum antimicrobial activity (Gram +ve, Gram −ve bacteria, fungi, schistosoma, parasites, and viruses) [6]. The antiviral effect of TQ was investigated in the murine cytomegalovirus model, and the results show that it enhances serum interferon-gamma levels and increases the numbers of CD4+ helper T cells [61]. Therefore, TQ has potential therapeutic applications for diverse pathological conditions, such as antiviral, anticancer, anti-inflammatory, and hepatoprotective interventions (Figure 2).

Along with this it also suppresses the function and facts of macrophages [6]. Another in vivo study reported that the TQ encouraged an extraordinary antiviral effect against murine cytomegalovirus contagion [24]. Many studies provide us with a solid backbone that either the plant *N. sativa* and/or TQ is responsible for the antiviral effect in different experimental models viz., in silico, in vivo, and in vitro. Various antiviral activities can be seen in Table 1.

## 5. Biopharmaceutical Problems of Thymoquinone

Viral infections are a global health challenge and SARS-COV-2 is the most recent addition to the list of global pandemic outbreaks [66]. Combating viral infections is a big challenge owing to the advent of new viral strains and a high level of genetic variability. These are often resistant to available drug inhibitors, and vaccines, and thus, the need for a new antiviral agent is always urgent and much required in the pharmaceutical market. For any agent to be a good antiviral agent, it should have an effective virucidal mechanism and a broad spectrum of activity against the viruses, and a good safety profile for the users [56]. Nature is a vast source of medicinal compounds derived from diverse organisms, such as plants, bacteria, and animals. However, plants are an essential source of phytochemicals. These phytochemicals have immense potential as medicinal and curative agents. The compendiums of their uses are mentioned in various traditional and alternative systems of medicine. TQ is one such multi-targeted wonder molecule from nature. The USFDA has classified Nigella oil as Generally Recognized as Safe [67]. TQ (2-isopropyl-5-methylbenzo-1,4-quinone) is the main active constituent of the *N. sativa* plant. The diverse therapeutic benefits of TQ viz. anti-inflammatory, antioxidant, anticancer, hepatoprotective, gastro-protective, antimicrobial, and anti-diabetic are well documented in both in-vitro and in-vivo. [68].

Despite being a wonder molecule of nature, TQ suffers from challenges related to poor biopharmaceutical characteristics and photo-instability. This hinders the potential of TQ, and it has not yet advanced to the clinical trial phase. TQ is a highly hydrophobic agent that has poor aqueous solubility issues [69]. The highly thermolabile nature of TQ, lack of quantification methods in blood and tissues, high hydrophobicity, and high lipophilicity (*Log P* = 2.54) causes poor formulation characteristics [70]. It is difficult to formulate TQ into formulations (tablets and capsules). In general, the use of herbal drugs in pharmaceutical research and development (R&D) is increasing exponentially in past few decades. The key benefits when utilizing natural prodrugs are that they are stable, economical, and have flexible properties; nevertheless, their main drawbacks include moderate water-solubility, poor oral bioavailability, limited half-life, and non-specific targeting problems. Nanotechnology provides a wide variety of potential solutions to tackle these challenges [71].

## 6. Nanotechnological Approaches of Thymoquinone for Effective Antiviral Therapy

**Figure 3:**
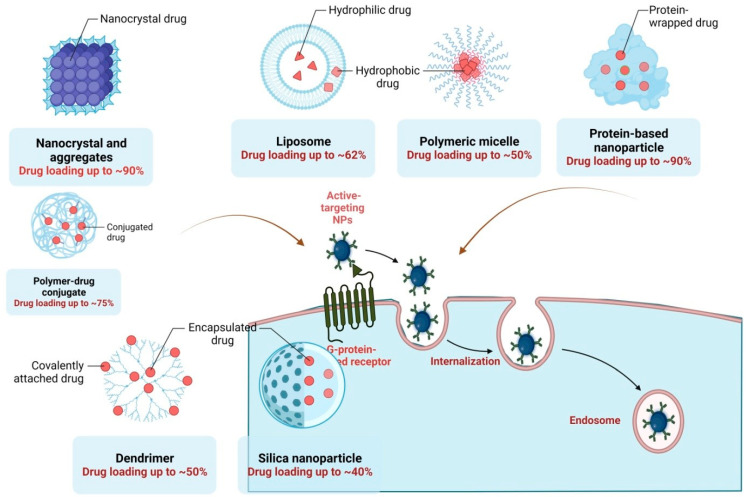
Illustration showing nano-formulations used as antivirals along with the drug loads they require.

Infectious diseases are a global burden, such as the unprecedented outbreak of SARS-COV-2 cases left the world crippled with no specific drug or vaccine to treat or prevent it immediately leading to high mortality. Since then researchers are working hard to explore, and identify more and more antiviral agents from natural sources and develop suitable nano-based formulations as antiviral therapeutics. The nanoparticles can address antiviral resistance a problem often associated with the conventional therapeutic approach by their unique properties such as small size, shape, advanced biological and functional properties, large surface-to-volume ratio, surface charges, and surface functionalization characteristics [72]. Nanomedicine by nanotechnology aims to create nanostructures to carry and target nanodrugs effectively. Many nanomaterials have been extensively researched in this regard to prevent viral diseases via the direct association of nanoparticles and viruses. The small size, big surface area, variety of surface modification, and ability to encapsulate drugs with large payload enhances the antiviral efficacies of these nanomaterials. Known for their unique physical and chemicals properties and used quite often for developing novel antiviral agents, recent research has categorized nanomaterials into four types (quantum dots as antiviral agents), metal nanoparticles (gold, silver, zinc oxide, and cobalt nanoparticles as antiviral agents), graphene-based nanoparticles (graphene and its derivatives–graphene oxide and reduced graphene oxide as effective antiviral agents) and photodynamic therapy (PDT for the inactivation of microbes) is the latest advancement in the field [73]. A thorough understanding of the pathogenesis of the viral disease, the virus structure, proteins present on the surface of the virus, and the mechanism of virus entry into host cells are vital in understanding to further fabricate a nanoparticulate formulation that is both safe and effective in the treatment of viral diseases. For a better understanding of the readers, researchers have systematically explained an approach focused on nanotechnology to tackle emerging NIPAH virus infection [74], the antiviral potential of silver nanoparticles against novel coronaviruses have also been addressed intricately by researchers [72], and antiviral potential of silver nanoparticles against African swine fever virus [75], Iron oxide nanozyme catalytically inactivates influenza virus [76]. Various forms of nano-formulations such as inorganic nanoparticles (gold nanoparticles, silver nanoparticles titanium oxide nanoparticles, carbon dots), organic nanoparticles, vesicular delivery systems, and Lipid-based nanoparticles, nanostructured lipid-based structures have been studied for the management of viral infections [77]. Metal nanoparticles’ promise as an auspicious therapy for viral and arboviral outbreak has been well documented (Figure 3) [78].

Nanotechnological approaches of different types are required to be applied for encapsulating TQ and improving its oral bioavailability and thus its efficacy. Numerous nano-formulations of TQ have been made in past for improved drug delivery in various human ailments such as Alzheimer’s disease, anticancer, hepatotoxicity [79,80]. The promise of this nutraceuticals as nano-TQ is comprehensively summarized in the literature, along with the benefits and drawbacks of other FDA-approved nanoparticle-based therapeutics [81]. Low water-solubility, poor oral bioavailability, poor penetration in membranes, and non-existence of understanding of its mechanism of action are some of the biopharmaceutical problems associated with TQ and all these are valid reasons behind the development of novel nano-TQ formulations with improved bioavailability [82]. In this section, we have tried to summarize the most novel and recent nano-TQ formulations with their characteristic features and improved profiles. TQ-loaded liposomes have been prepared in past for improved stability, bioavailability, and enhanced anticancer activity towards breast cancer [83]. TQ-loaded NLCs were prepared using high-speed homogenization accompanied by ultrasonication, and their in-vitro properties were assessed. TQ-NLCs have higher relative bioavailability than TQ suspension, meaning that the NLC formulation improves bioavailability [84]. Poor oral bioavailability upon oral administration impedes TQ biological activity. TQ-SNEDDS were created by scientists and evaluated for improved hepatoprotective potential. As compared to TQ suspension, optimized SNEDDS formulation of TQ showed improved in-vivo absorption and an increase in relative bioavailability up to 3.87 folds [85]. Modified chitosan-loaded TQ nanocapsules were also prepared recently by the researchers. The optimized nanocapsules had a size between 100 and 300 nm and a zeta potential of +48 mV indicating a more stable profile [86]. A mitochondria-targeted TQ compound was produced in an extremely advanced and novel manner, mitochondrial function in rat liver and yeast cell viability were both studied. Improved antioxidant activity of SkQThy was observed showing good therapeutic potentials [87]. The anticancer potential of TQ has been enhanced by various nanotechnological approaches and researchers have reviewed various TQ-nano-formulations highlighting their superior anticancer efficacy such as in breast cancer [71,80]. In yet another exciting study, inclusion complexes of TQ and hydroxypropyl-β-cyclodextrin were prepared for improved solubility and bioactivity. As compared to free TQ, the complexed TQ had better improved efficacy against allergies and prolonged action with reduced side effects [88]. Non-lamellar liquid crystalline nanoparticles made up of cubosomes and hexosomes are gaining popularity as robust nanocarrier systems for TQ due to their special properties. The lipid composition and drug loading of these nano-self-assemblies have considerable impact on their structural features, morphological characteristics, and size. Cubosomes and hexosomes are vesicular nanostructures possessing “flower-like” features that are ideal for enhancing the solubilization capacity of poorly water-soluble drugs and improving drug delivery through various routes of drug administration.

Recently, nano-dispersions, comprising binary mixtures of glycerol monooleate and vitamin E loaded with TQ, were prepared and characterized by their morphological, structural, and size characteristics [70]. Very recently, TQ-loaded NLCs were once again formulated to improve their poor solubility and oral bioavailability issues. A high-pressure homogenization method was employed, and a particle size of less than 100 nm was achieved for the nanocarriers, which showed stability for up to 24 months of storage. The formulated nanocarrier of TQ was radio-labelled with technetium-99 m before administration to the rats for the in-vivo organ distribution study both by oral and I.V. routes. TQ-NLCs administered orally had a higher relative bioavailability than those administered intravenously, but oral administration had a slower absorption rate than intravenous administration [89]. In yet another recent study, TQ-loaded Soluplus–Solutol HS15 mixed micelles were prepared. In-vitro characterization was carried out and their effects on SH-SY5Y cell migration were studied. The use of nanotechnology to inhibit cancer cell invasion and migration has opened up new avenues for combating neuroblastoma [90]. MSNs (mesoporous silica nanoparticles) have gained much popularity in drug delivery systems in the last few decades for being an effective delivery system for targeted drug delivery. Their low toxicity profile, small particle size, uniform pore size, good biocompatibility, and chemical stability have made them ideal nanocarrier systems for drugs [91]. The mesoporous silica nanoparticles (MSNs) (nanospheres with a size of 100–200 nm in diameter) are known to enhance the solubility profile of drugs. In addition, they have an excellent targeting ability and are capable of loading two or more drugs for dual therapeutic efficiency. These are biocompatible, non-toxic, and also classified as “Generally Recognized as Safe” (GRAS) by the FDA. In a very recent study, MSNs were used as nanocarriers of natural antiviral prodrug complexes of shikimic acid and quercetin. The developed novel nano-formulations were highly effective against the influenza virus H5N1 [92]. A similar approach can be applied to TQ for improving its antiviral efficacy. To further surmount the delivery challenges and biopharmaceutical issues associated with TQ, researchers are working on various other nanosystems. Very recently, phospholipid nanoconstructs were constructed by Rathore et al., 2020 [93]. The optimized nano composition had a size of <100 nm, a spherical morphology (nanospheres), entrapment efficiency of >90%, polydispersity index of 0.55, a controlled-release pattern, and a zeta potential of −0.65 mV. The single dose, upon oral administration, produced a relative bioavailability of 386.03% and improved the hepatoprotective effects as compared to a plain TQ suspension. This nanoconstruct has the potential to be a promising delivery mechanism for increasing the oral bioavailability of this hydrophobic compound. The nanosizing of phytochemicals whose clinical usefulness is restricted due to poor biopharmaceutical attributes is also a novel approach to enhancing their therapeutic efficacy. The nanosizing of curcumin, naringenin, berberine, catechins, TQ, resveratrol, apigenin, baicalin, and many other molecules is evident from the literature [81]. Recently, TQ-loaded SLNs were prepared and tested in rats for their antidepressant activity [94]. Keeping in mind all of the biopharmaceutical issues of TQ, the ideal approach is encapsulation or entrapment of this bioactive compound in a vesicular system such as nanoliposomes and tocosomes, which have greater potentials as advanced vesicular systems of drug delivery. The encapsulation of nutraceuticals improves their stability profile, controls their release characteristics and increases the product shelf life [95]. TQ-loaded cubosomes with a mean particle size of 98 ± 4.10 nm, entrapment competence of 96.60 ± 3.58%, and zeta potential of 31.50 ± 4.20 mV were prepared recently using the emulsification homogenization method. They were evaluated for their in-vitro chemotherapeutic efficacy on MCF-7 and MDA-MB-231 breast cancer cell lines, compared to MCF-10A non-tumorigenic cell lines. The enhanced anti-tumor activity of TQ-loaded cubosomes was observed as compared to free TQ [96].

A nano-based approach to combat viral infection using natural phytoconstituents (TQ) is a new arena and more research is warranted. Nano-TQ-based formulations seem to be a versatile and feasible strategy to conquer viral infections due to the potent antiviral properties of TQ. A well-designed and targeted nano-TQ system against the viral proteins that facilitate the spread of infections could help prohibit the spread of viral infections. A nano-TQ-based antiviral nanomedicine with improved antiviral activity is a prerequisite in this regard to protect mankind from these rapidly emerging viral infections. With the nano-based formulation approach, it is much easier and more promising to bridge the gap between the occurrences of disease, a cure, and improved and biomedical applications of agents. Nanoproducts with improved bioavailability, bio-degradability, biocompatibility, tunability, targetability, and specificity are the expected outcomes in this regard. Thus, more and more nanotechnological approaches could be applied to make more nano-TQ formulations with improved bioavailability and enhanced antiviral efficacies in the near future.

## 7. Current Clinical Trials and Patents of Thymoquinone with Antiviral Potency

Natural supplements have various confirmed effects on human health, ranging from immune system stimulation to effective antiviral and antioxidant activity. Omega-3 also has an antiviral effect on the influenza virus, preventing it from replicating. On the other hand, black seed supplementation has a chelating role in patients with sickle cell anemia and inhibits human heme metabolism. Furthermore, *N. sativa* and its active constituent TQ inhibit the replication of SARS-CoV-2 [97], as well as the expression of members of the TRP-gene family (NCT04553705) [98]. Anti-inflammatory, antioxidant, anti-tumor, and antimicrobial effects are all found in TQ [98,99,100,101]. In a pilot study, children with refractory epilepsy were given TQ at a dose of 1 mg/kg as an adjunctive treatment. The effects on seizure frequency were compared to those of a placebo. The study enrolled twenty-two patients. They were only given their pre-existing anti-epileptic medication during the two-week washout period. For four weeks, they were divided into two groups and given either TQ or placebo. They were then given TQ or a placebo for another four weeks. In infants with refractory seizures, TQ appears to have anti-epileptic properties [102]. Moreover, eighty-one patients (aged 18 to 75 years) with oral potential premalignant lesions participated in this randomized controlled trial. Patients were divided into three equal groups at random to receive *N. sativa* 10 mg buccal tablets, group A; *N. sativa* 5 mg buccal tablets, group B; or placebo or control (group C). A summary of the clinical trials are presented in Table 2.

The relevant and recent patents related to TQ as a bioactive carrier in viral infections have been concisely presented in this section. Table 3 contains a synopsis of the innovation as well as the medicinal benefits for the readers’ convenience. The recent patents enlisted here show this approach’s viability for delivering TQ, the antiviral drug, to their required targeted site of action. The increased potency of TQ loaded in various carrier systems suggests that this novel drug delivery system will become more widely used in antiviral therapy.

## 8. Advancements in the Field

TQ is a phytochemical agent that can be used to cure several diseases. The main bioactive compound found in the *N. sativa* plant, TQ, is connected to most of the plant’s biological activities such as anti-cancer, anti-viral, and antioxidant activities and many more [101]. However, because of its low aqueous solubility and bioavailability, it has become difficult to use. TQ’s poor water solubility is one of the issues that limits its therapeutic efficiency. At room temperature, the solubility of TQ in water was found to be 1.0 mg/mL, so higher doses of TQ were required to achieve a therapeutic effect [84,88]. TQ has a poor dissolution rate and low water solubility, rendering it unsuitable for oral administration due to its low bioavailability. TQ’s biopharmaceutical problems restrict its use as an ideal drug candidate for oral administration, such as its pH instability, photo-degradability, high hydrophobicity, high first-pass metabolism, and poor systemic bioavailability. TQ has weak formulation properties due to its hydrophobic chemical nature. It has poor membrane penetration in humans [110]. The use of curcumin (CUR) and thymohydroquinone (THQ) in conjunction may have a more significant therapeutic benefit [102,111]. CUR and THQ have been shown to exhibit significant effects even low doses [112]. Temperature and light are two environmental variables that influence TQ. The use of nanoliposomes or nanoscale bilayer lipid vesicles may be a solution to these problems. Nanoliposomes distribute therapeutic agents in a controlled way, at the right time and in the right place. Nanoliposomes are smart drug delivery systems with a high biocompatibility index as they are composed of lipids and phospholipids. They have a greater surface area, and a good stability profile, nanometric size, and amphiphilic nature to entrap both hydrophilic and hydrophobic drugs simultaneously for combined effects. Regardless of their solubility, they improve the encapsulated material’s potency and cellular absorption. Nanoliposomes revolutionize reactive, volatile, or responsive molecules into stable ingredients [20].

## 9. Future Prospects

The future prospects for nano-formulations of thymoquinone (TQ) as a potent natural antiviral agent are promising. With various ongoing viral infections, there is an urgent need for effective antiviral drugs, and TQ has shown potential for use in the treatment of viral infections. Nano-formulations of TQ can further enhance its pharmacokinetic and bioavailability properties, leading to improved antiviral activity and potentially better clinical outcomes. One area of future research could be the development of targeted nano-formulations of TQ [79,80]. Targeted nano-formulations can selectively deliver TQ to the site of viral infection, resulting in higher concentrations of the drug at the target site, potentially reducing the risk of side effects. Targeted nano-formulations can also improve the therapeutic index of TQ, allowing for higher doses to be administered without toxicity. Another area of future research could be the investigation of combination therapy using TQ and other antiviral agents. Nano-formulations of TQ could be combined with other antiviral drugs to enhance their efficacy and reduce the risk of drug resistance. For example, TQ has been shown to enhance the antiviral activity of ribavirin against HCV, and nano-formulations of TQ could potentially improve the efficacy of other antiviral drugs against a range of viral infections. In summary, the future prospects for nano-formulations of TQ as a potent natural antiviral agent are promising, and further research in this area could lead to the development of more effective treatments for viral infections.

## 10. Conclusions

TQ-derived from *N. sativa* has been used as a traditional medicine for thousands of years, and its wide range of medicinal applications in various ailments has resulted in researchers exploring its biological active compounds. TQ may play a potential role in the treatment of various diseases including viral infections, as a result of its immunomodulatory, anti-inflammatory, and anticancer effects. Currently, different types of viral diseases have spread in the community; among these, some have presented serious global threats. In this regard, TQ obtained from *N. sativa* might be a promising natural alternative to cure viral infections, particularly against SARS-CoV-2, due to its antiviral action. It has the potential of binding SARS-CoV-2 and has the potential to disrupt virus–host interactions. The antiviral action of TQ is illustrated by it modulating the production of NO and ROS and reducing a cytokine storm. However, it is difficult to formulate TQ into formulations such as tablets and capsules. There is an urgent need to search for a substitute that could produce a superior outcome and that is easy to formulate. Consequently, nano-formulation is an alternate approach to achieve a better antiviral efficacy and improve bioavailability. Furthermore, studies and clinical trials are being conducted to discover antiviral and immunomodulatory activities that could be a promising potential approach for treating SARS-COV-2 and other viral diseases.

## Figures and Tables

**Table 1:** Various antiviral activities of Thymoquinone (TQ).

S.No	Microorganism	Treatment	Experimental Model/Assay	Results	Ref.
1.	Peste des petits ruminant’s virus (PPRV)	Vero cell lines infected with PPRV were treated with six dilutions of *N. sativa*	MTT assay and Plaque reduction assay	All 3 dilutions (50, 25, 12.5 µg/mL) exhibited significant antiviral activity with a significant reduction in plaques count.	[62]
2.	Murine Cytomegalovirus	*N. sativa* oil (100 µg/100 µL/mouse for 7 successive days)	The Murine Cytomegalovirus model was used for viral plaque forming assay, flow cytometry, cytolytic activity, ELISA assay, suppressor function assay, cytolytic T lymphocyte assay	Treatment with oil enhances the production of interferons-γ and causes augmentation in CD4+ helper T cell number, suppressor function, and facts of macrophages.	[6]
3.	H9N2 avian influenza viruses	4-week-old mixed-sex turkey poults	Characterization of cytokine gene expression, as well as the determination of whether or not virus particles are being shed and serological examination.	Improved cytokine gene expression and thus suppressing the pathogenesis of H9N2 avian influenza viruses	[48]
4.	Hepatitis C virus	*N. sativa* at 450 mg thrice a day	Liver markers enzymes, hepatitis B surface antigen, hepatitis B core immunoglobulin G, and Hepatitis C virus antibody.	Enhanced oxidative stress and diminished viral load.	[24]
5.	Coronavirus	Thymoquinone dissolved in Dulbecco’s Modified Eagle’s Medium	In-silico, cytotoxicity, and plaque reduction assay	Enhances the binding opportunity of thymoquinone with the main protease (M^pro^).	[63]
6.	*Plasmodium yoelii*	Methanolic extract of *N*. *sativa* seeds (1.25 g/kg)	in vivo model of malaria on Swiss albino mice	Mice that had been infected had their oxidative state in their red blood cells and hepatocytes illuminated.	[23]
8.	*Eimeria stiedae*	400 mg/kg *N. sativa* aqueous and oil emulsions	*Eimeria stiedae* infection in rabbit	Shows anti-coccidial effects with a rapid antiparasitic consequence	[64]
9.	Coronavirus (SARS-CoV2)	In-silico inhibition of the replication by thymoquinone	In- silico model	SARS-CoV2 protease-inhibiting properties	[65]
10.	Human cytomegalovirus	Thymoquinone bis artesunic acid hybrid	In-vitro inhibitory activity against Human cytomegalovirus in human fibroblasts cells.	The hybrid compound is more potent than ganciclovir and artesunic acid (EC_50_ 0.63/0.56/0.69 M).	[46]

**Table 2:** A detailed summary of clinical trials related to thymoquinone:

Clinical Trial.Gov Identifier	Study Title	Purpose	Status	Sponsor	Study Type	No of Participants	Allocation	Outcomes
NCT03208790	Clinical and immunohistochemical evaluation of the chemopreventive effect of thymoquinone on oral potentially malignant lesions.	Treatment	Completed	Cairo University	Interventional	30	Randomized	The results of the study have not been posted until now.
NCT04686461	Effect of thymoquinone extracted from *Nigella sativa* in the treatment of arsenical keratosis.	Treatment	Recruiting	Bangabandhu Sheikh Mujib Medical University, Dhaka, Bangladesh	Interventional	34	Allocation: N/A	Keratotic nodular size
NCT03776448	The effect of 2 g daily supplementation of *N. sativa* oil on blood glucose levels of adults.	Treatment	Unknown	Sulaiman AlRajhi Colleges Al Bukairiyah, Qassim, Saudi Arabia	Interventional	30	Randomized	Fasting venous blood glucose, blood pressure, gastrointestinal symptoms
NCT04553705	Omega-3, *Nigella sativa*, Indian costus, quinine, anise seed, deglycyrrhizinated licorice, artemisinin, and febrifugine on the immunity of patients with SARS-COV-2	Treatment	Recruiting	Maternity and Children’s hospital Mecca, Makkah, Saudi Arabia	Interventional	200	Randomized	Clinical improvement, The recovery rate from positive to negative swabs, and fever to normal temperature in days.
NCT04292314	Hydroxyurea, omega 3, *Nigella sativa*, and honey on oxidative stress and iron chelation in pediatric major thalassemia	Treatment	Completed	Beni-Suef University	Interventional	350	Randomized	F 2-isoprostanes pg/mL, HDL cholesterol Mg/dL.

**Table 3:** The relevant and recent patents related to TQ as bioactive carriers in viral infections.

Patent Number	Invention Title	Description of the Invention	Pharmaceutical Advantages	Ref.
WO2016005786	Liposomal formulations comprising thymoquinone and taxane, and methods of treating cancer using the same.	This disclosure relates to liposomal pharmaceutical compositions comprising taxane and thymoquinone.	Significantly enhances the stability of the liposomes and provides more consistent taxane release patterns.	[20]
WO2016061117	Nano-liposomal aminoglycoside–thymoquinone formulations	The liposome-encapsulated aminoglycoside–thymoquinone (TQ) formulations can be administered to a subject in need.	The liposome-encapsulated aminoglycoside–thymoquinone formulations enhanced the efficacy of thymoquinone	[103]
US9745242B1	A method for the production of thymoquinone	A method for producing thymoquinone and thymohydroquinone in Monarda.	Monarda with elevated levels of carvacrol and thymol in the fresh plant tissue and vigorous growth.	[104]
WO2013172537A1	Pharmaceutical composition for treating or preventing neurological disorders caused by alcohol exposure during pregnancy, containing metformin and/or thymoquinone	This invention relates to a pharmaceutical composition for treating or preventing neurological disorders caused by fetal alcohol exposure	The combination is effective in preventing or treating neurological disorders caused by alcohol exposure of a fetus.	[105]
KR101493139B1	Composition for protecting nerve cells which comprise thymoquinone and vitamin C as active ingredients	This invention relates to a composition for protecting nerve cells, a composition for preventing or treating nerve disorders, and a healthy functional food for preventing or improving nerve disorders.	The composition of the present invention, which simultaneously comprises thymoquinone and vitamin C, has the effect of protecting nerve cells.	[106]
CN111000836B	Application of thymoquinone and combined use of thymoquinone and autophagy inhibitor ATG7-siRNA in the preparation of drugs treating esophageal cancer	The invention relates to the application of thymoquinone TQ in preparation for a composition treating esophageal cancer.	The inventor also found that the combined use of an esophageal cancer cell autophagy promoter siRNA and TQ had the characteristic of synergistic inhibition of esophageal cancer cell proliferation.	[107]
US8895625B2	Protective effect of thymoquinone in sepsis	This invention refers to thymoquinone, a primary constituent of the volatile oil of *Nigella sativa*, and its protective effect against sepsis syndrome morbidity, mortality, and associated organ dysfunctions.	This invention refers to thymoquinone for use in the prevention and/or treatment of sepsis syndrome. This invention further refers to a pharmaceutical composition and a kit.	[108]
WO2013030669A4	Compositions comprising thymoquinone for the treatment of inflammatory diseases	The invention provides a method of treating at least one symptom of an inflammatory disease or disorder in an individual in need of such treatment; the method comprises administering an effective amount of thymoquinone to the individual.	Thymoquinone and at least one physiologically acceptable carrier, wherein an effective amount of thymoquinone could reduce or prevent at least one symptom of the inflammatory disease or disorder.	[109]

## Data Availability

Not applicable.
